# Methods for measuring horizontal equity in health resource allocation: a comparative study

**DOI:** 10.1186/s13561-014-0010-x

**Published:** 2014-08-10

**Authors:** Yi Tao, Kizito Henry, Qinpei Zou, Xiaoni Zhong

**Affiliations:** School of public health and management, Chongqing medical University, 1# yixue Rd., Chongqing, 400016 China; Faculty of Health, Medicine and Life Sciences, Maastricht University (the Netherlands), Leuven, 3000 Belgium

**Keywords:** Methodology, Equity, Health resource allocation, Health resource

## Abstract

**ᅟ:**

There are many existing methodologies on measuring health equity, while seldom has method aiming at health resource allocation. We collected 6 method of measuring equity in health resource allocation. This paper presents key contents of methods on measuring horizontal equity in health service allocation, yet each method has its advantages and disadvantages as well as range of application, which may help researchers or government to make wise decision when choosing appropriate method for measuring equity. Through comparative analysis, we concluded that socioeconomic factors were considered in concentration index; although the Lorenz curve and Gini-coefficient are widely used, which exist uncertainty and incompleteness; overall inequality can be decomposed by Theil index, which is of significance for the planning of urban and rural areas; preferences on a certain class can be set artificially by Atkinson index; it is easy for Chi-square to analyze aided with statistical software; specific regional differences can be calculated by index of dissimilarity.

**Classification codes:**

I1

**Electronic supplementary material:**

The online version of this article (doi:10.1186/s13561-014-0010-x) contains supplementary material, which is available to authorized users.

## Introduction

Health resource allocation refers to the health resource which were distributed and flowed among health care industry (or departments), and also influenced by the factors as convenience level for medical service; hierarchy of needs and quantity; the quantity, quality, and scope of supply which could be actually provided by medical and health organization; and effective utilization degree etc. [1],[2].

From the view of [3],[4], the equity in the realm of health resource means the distribution of health resources should be based on the needs as the orientation, rather than depending on the social privilege or income difference; should share the results of social progress, rather than sharing inevitable misfortune and loss of health right [5]. Obviously, equivalent health service can hardly meet the need of every individual, which will lead too little health services utilization coexists with too much. Managing health resources and health care effectively and efficiently is an important part of promoting the development of public health. Experience has shown that, without strategic policies and focused spending mechanisms, the poor and other ordinary people are likely to get left out [6].

The issue of health resource allocation has become more and more concerned to scholars. Almost all scholars agree that equity in health resource is divided into vertical and horizontal dimensions. Horizontal equity refers to the social members who have equal need for health resource receive the same [7]; vertical equity emphasizes individuals with different levels of need can receive appropriately different amounts of health resources ([8]).

There are many methods for measuring health equity or equity first applied in the realm of economy, however, seldom has method aiming at health resource allocation. Under the consideration of simpleness, commonness and easy-comprehension, this paper summarized six methods for horizontal health allocation equity estimation, each method has its advantages and disadvantages, as well as applicable conditions, and been analyzed through definition, calculation method, application, data requirement and other aspect. Meanwhile, different research methods are also likely to produce different results.

## Review

### Methodology of measuring horizontal equity

In health resource delivery, inequity is means that discrimination for non-need factors, since we determined that only allocation according to need is equitable [9]. An easy method to test for the existence of inequity in health resource allocation is to test whether two (or more) groups (for instance the rich and the poor or different regions) receive the same amount of resource [10]. When we compare whether inequity is present, we need to take into account whether these two groups have the same amount of need (are equal) and therefore whether are completely comparable. This can be amended by correcting for the difference in need between the groups, either via direct or via indirect standardization method. They can be compared, when both groups have equal needs, or when standardized to equal need.

### Method of concentration curve and concentration index

#### Concentration curve

The concentration curve [11],[12], and related concentration index (CI), have now days attained the status of “workhorse” in most health economic studies” [13]. For example, it could be used to assess whether subsidies to the health sector are well targeted towards the poor among countries [14], or whether inequalities in health resource allocation are more pronounced in some countries than in others [15]. And other applications are also possible. When examining the equity of health care resource allocation, it uses the concept of horizontal equity, i.e. treating people with equal need the same and irrespective of their income [16].

**Figure 1:**
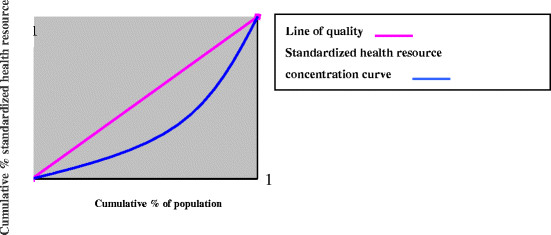
**Standardized health resource concentration curve.**

The concentration curve plots the cumulative percentage of the health resource variable (y-axis) against the cumulative percentage of the sample, ranked by living standards, beginning with the poorest, and ending with the richest (x-axis) (See Figure 1 below for examples of concentration curve). For example, the concentration curve might show the cumulative percentage of exp. accruing to the poorest p% of the sample. If everyone, irrespective of his living standards, has exactly the same value of the health resource variable, the concentration curve will be a 45°line, running from the bottom left-hand corner to the top right-hand corner. This is known as the line of equality. If, by contrast, the health resource sector variable takes higher(lower) values amongst proper people, the concentration curve will lie above(below) the line of equality. The further the curve is above the line of equality, the more concentrated the health variable is amongst the poor. If the variable takes on smaller values amongst the poor, the concentration curve will lie below the line of equality, and the further below the line of equality, the more concentrated amongst the better off the variable in question is [17].

#### Concentration index

The concentration index is an index to investigate the unfair degree of a certain variable associated with social and economic status, which dynamically reflects the effect of the variable influenced by income.

The concentration index is defined with reference to the concentration curve(q.v.), and defined as twice the area between the concentration curve, g_exp_ (Figure 1), and the line of equality (the 45°line running from the bottom-left corner to the top-right). So, in the case where there is no income-related inequality, the concentration index is zero [17]. When computing, firstly, rank by social class with corresponding rank (X); then calculate a certain variable level (H) of one social class level, and according to this variable mean (M), can finally work out CI.

1 $$\mathrm{CI}=\;\frac{2\times \mathrm{COV}\left(X,H\right)}{M}$$

2 $$\mathrm{COV}\left(X,H\right)=E\left(\mathrm{XH}\right)-E\left(X\right)E\left(H\right)$$

Where, COV(XH) is the covariance of (X) and (H), E (XH) is the mathematical expectation of the product for (X) and (H), E(X) is the mathematical expectation of (X), E(H) is the mathematical expectation of (H).

C lies in range (−1—1). CI < 0, indicates health resource variable is disproportionately concentrated on poor; CI > 0, indicates health resource variable is disproportionately concentrated on rich; C = 0, when distribution in proportionate; the further the CI deviate from 0, the higher level of unfair will be. C = 1, if richest person has the entire health resource variable; C = −1, if poorest person has the entire health resource variable.

### Method of Lorenz curve and Gini-coefficient

#### Lorenz curve

**Figure 2:**
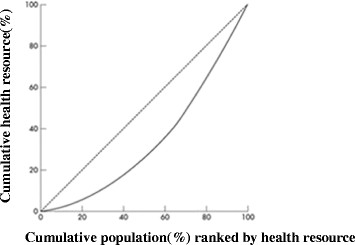
**Lorenz health resource curve.**

The Lorenz-curve was first developed by Max O. Lorenz in 1905, as a graphical representation of income distribution. In the field of health, Lorenz curve is a way to measure horizontal equity, the x-axis of which represents the cumulative proportion of individuals by level of health resource, ranked in increasing order—that is, beginning with the persons with the least resource and ending with those who are with the most; while the y-axis represents the cumulative total proportion of health resource of relative region (Figure 2). If health resource is equally distributed among individuals, the Lorenz curve is a diagonal line. The more it deviates from the diagonal, the larger the degree of allocation inequality.

#### Gini-coefficient

As a foundation of welfare economics to measure inequity in health resource, the primary measure of income inequality, Gini-coefficient (GINI), has been widely used to test the relationship between inequality and health [18],[19]. A region with no inequality will have a value of 0 while a region with complete inequality will be denoted by a Gini-coefficient of 1. Given a Lorenz-curve plot, we can measure the degree of inequality of the distribution of health resource by a one-dimensional number, the so-called Gini-coefficient ([8]).

**Figure 3:**
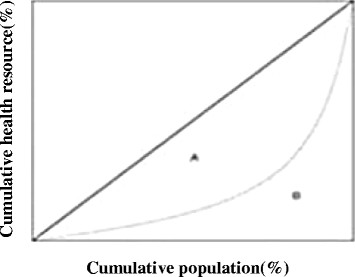
**Lorenz curve.**

The Gini-index, which is twice the area between the Lorenz curve and the equiangular line, which could be calculated as follows (Figure 3).

[1] GINI-coefficient = Area A/(Area A + Area B)

The higher the GINI-coefficient is, the more unequal is the resource being distributed across the population in question.

Because 100% is equal to 100/100 = 1, and the two axes in the Lorenz curve goes from 0% to 100%, the area of the entire box must be 1. It follows that Area A + Area B must equal ½.

The Gini-coefficient therefore also can be written as,

[2] Gini-coefficient = (Area A)/(1/2) = 2 × (Area A)

It is the metric you see when Gini-coefficients are shown.

The Gini-coefficient ranges from 0 to 1, with 0 signifying perfect equality (the Lorenz curve coincides with the diagonal line in Figure 3) and with 1 signifying perfect inequality. The standard of Gini-coefficient in health resource allocation refers to income distribution fairness in economics. Gini-coefficient < 0.3, indicates in perfect equity condition; 0.3-0.4, indicates in normal condition; >0.4, indicates in alert condition, >0.6, indicates in highly inequity perilous condition [20].

#### Theil index

Theil index is first used by Dutch economist H. Theil, who uses the entropy concept to calculate the unfairness of income, TI range from [0, 1], the smaller the TI value is, the more unequal between regions will be. Although the Theil Index (TI) was originally proposed as measures of income inequality [21]-[25], it is now common measures of disparity in health research [26]-[28] and of inequity in health resource allocation, because it is decomposable by groups, and can incorporate group-level data and is particularly effective at paring effects in hierarchical data sets. The formula is as follows;

3 $$T=\sum_{i=1}^{n}P_{i}\log\frac{P_{i}}{Y_{i}}$$

In formula 3, *P*_*i*_ represents the proportion of some place’s population accounts for total population; *Y*_*i*_ represents the proportion of health resources owned by some place accounts for the total number of health resources. A weighted sum of inter-unit inequality within each group, called the “within group” component, and a “between-group” component that measures inequality due solely to variations in health resource density across groups. The decomposition formula is;

4 $$T=T_{\mathrm{intra}-\mathrm{class}}+T_{\mathrm{inter}-\mathrm{class}}$$

5 $$T_{\mathrm{intra}-\mathrm{class}}=\sum_{g=1}^{k}P_{g}P_{g}$$

6 $$T_{\mathrm{inter}-\mathrm{class}}=\sum_{g=1}^{k}P_{g}\log\frac{P_{g}}{Y_{g}}$$

In the above formulas, ‘T_intra-class_’ in this article means the differences of health resource allocation in the area; ‘T_inter-class_’ means the differences of health resource allocation between areas; *P*_*g*_ represents the proportion of some place’s population accounts for total population; *Y*_*g*_ represents the proportion of health resources owned by someplace accounts for the total number of health resources. The contribution rate of the difference between each part on total theil index can be calculated after decomposing the theil index. For health resource allocation, if the TI = 0, means equity in allocation, the smaller the value is, the more equity in allocation will be, and vice versa.

#### Atkinson index

The Atkinson index is an index used to assess income inequality which was developed by a British economist known as Anthony Barnes Atkinson. The measure is useful in determining which end of the distribution contributed most to the observed inequality [29]. The Atkinson index has a specific feature for the calculation of distribution. The index uses the epsilon parameter to explicitly reveal the inequality aversion of allocation. Epsilon defines how sensitively the Atkinson index should react to inequalities. The Atkinson index is an inequality measure based on health resource allocation, which defines maximum inequality as 1 and maximum equality as 0 [30]. The definition is as follows,

7 $$I_{R}=1-{\left[\sum_{i=1}^{n}{\left(\frac{Y_{i}}{\bar{Y}}\right)}^{1-\varepsilon }f_{i}\right]}^{\frac{1}{1-\varepsilon }},\mathrm{if}\;\varepsilon \neq 1$$

8 $$I_{R}=1-\exp\left[\sum_{i=1}^{n}f_{i}\log_{e}\frac{Y_{i}}{\bar{Y}}\right],\mathrm{if}\;\varepsilon =1$$

*ε* is a parameter related to the external clearer display of regional imbalance, called inequality aversion. The parameter reflects social equal degree for inequality aversion (or preferences). 0 < *ϵ* < + ∞, the higher the *ε* is, the display of imbalance will be more obvious, when *ε* = 2, Atkinson index can reveal moderate imbalance. For assessing equity of health resource, *Y*_*i*_ is the health resource gotten by individuals in the ith health resource range (N ranges altogether), *f*_*i*_ is the proportion of the population with health resource in the ith range, $\bar{Y}$ is the mean health resource in group.

For assessing the equity of health resource allocation, an intuitive interpretation of this index is possible: Atkinson values can be used to calculate the proportion of total health resource that would be required to achieve an equal level of allocation as at present if health resources were perfectly distributed. For example, an Atkinson index value of 0.20 suggests that we achieve the level of allocation with only 1–0.20=80% of health resource. The theoretical range of Atkinson values is 0 to 1, with 0 being a state of equal distribution [31]. The smaller the index is, the more equal the allocation will be, and vise verse.

#### Chi-square value method

It was statistician Pearson (1857–1936), who first proposed chi-square test (X2 test). As one of the nonparametric tests, X2 test is a significance test to compare the difference of two or more sample rates (or ratio), the theoretical basis of which is Continuous probability distribution (X2 distribution). By comparing two or more frequencies, detecting the difference between the actual frequency and the expectation frequency based on distribution hypothesis in a certain significance level, which reflects whether there exists significant difference between the actual level and theoretical level. The theory is as follows,(1) Computational formula,

9 $$X^{2}=\sum_{i=1}^{k}\frac{{\left(f_{i}-\bar{f_{i}}\right)}^{2}}{\bar{f_{i}}}$$

*f* i ( i = 1, 2, ,, k) means there are k actual frequencies, which can be gotten through the investigation and experiment; $\bar{f}$ i ( i = 1, 2, ,, k ) means there are k expectation frequencies, which should be calculated according to the statistical hypothesis or refer to relevant data.

(2) X2 test steps

The specific steps are stated as follows,

1. Establish the null hypothesis H_0_ and alternative hypothesis H_1_;
2. Calculate expectation frequency according to the theoretical distribution (or empirical distribution);
3. Calculate sample chi-square value according to the actual frequency and expectation frequency (formula 4);
4. Find corresponding chi-square critical value According to the degree of freedom and significance level a in the chi-square distribution list. If the calculated value is less than chi-square critical value, accept the null hypothesis; otherwise, accept alternative hypothesis.

When calculating, we should check whether variables are completely comparable, which can be amended either via the direct or the indirect standardization method.

(3) Evaluation of X2 value on equity of health service allocation

Analyze form the formula, the general term ${\frac{\left(f_{i}-\bar{f_{i}}\right)}{\bar{f_{i}}}}^{2}$ (*i =* 1,2…,k) is a ratio of the square of *f*_*i*_ deviated from $\bar{f_{i}}$ with $\bar{f_{i}}$, the sum of which reflects the difference between distribution of *f*_*i*_ and distribution of $\bar{f_{i}}$. The bigger the chi square value, the more significant difference between distributions will be, and vice versa. Hence, the variation of chi-square value can reflect variable trend of the difference degree between variables’ actual distribution and theoretical distribution. For health resource allocation, if the chi-square value less than critical value, indicates that in a certain significance level α, this kind of health resource is allocated fairly, and vice verse; from the perspective of the variable trend, by comparing a health resource variate in different years, which can also indicates the trend of the equity of health resource allocation.

#### Index of dissimilarity

Index of Dissimilarity (ID) expresses the extent to which the distribution of the health event studied in the population approximates the situation in which everyone has the same socioeconomic level [32]. In the field of health service allocation, This indicator can be applied to variables related to health resource, such as the number of physicians that would be necessary to redistribute among municipalities to achieve equity (Schneider et al. [33]); ID also can judge whether there are differences in health service allocation between regions, and the degree of differences between regions can be calculated. The definition of ID is as follows;Suppose there are j = 1, 2….k different regions (different socioeconomic levels), index of dissimilarity is;

10 $$\mathrm{ID}=\frac{1}{2}\sum_{j=1}^{k}\left|S_{\mathrm{jh}}-S_{\mathrm{jp}}\right|$$

*S*_*jh*_ means the proportion of a certain variable which can reflect the equity of health resource allocation of jth region (or jth region of a certain socioeconomic level); *S*_*jp*_ means the population proportion of jth region (or in a certain socioeconomic level). The greater the differences between *S*_*jh*_ and *S*_*jp*_, the higher the health resource inequality degree is. The ID value is between 0 and 1, if the ID = 0, means equity in allocation, the smaller the value is, the more equity in allocation will be, and vice versa. The index of dissimilarity is large, when large parts of the population are in low and high socioeconomic groups and there are few people in intermediate groups [32].

## Conclusions

Table 1 summarizes the measures mentioned in this paper and makes some recommendations about the use of the various measures.

**Table 1:** **Summary of health resource allocation equity measures**

Measure	Definition	Complexity of calculation	Application	Required data	Benefits	Caveats
Concentration curve, concentration index	Calculate and compare cumulative percentage of population (ranked by socioeconomic factors) and health resource. CI $\mathrm{CI}=\;\frac{2\times \mathrm{COV}\;\left(X,H\right)}{M}$_,_ COV(X,H) = E(XH)-E(X)E(H)	Complex but aided by statistical software	Systematic assessment, and can be a rough estimation on equity of differences between different regions.	Income of individual, health resource of individual	-not only represent overall inequity, also reflect accurately which social classes allocated with more resource and which less via positive or negative CI value	-incapable of considering the other variables, especially the resource delivery itself.
					-socioeconomic factors are taken into consideration when measure the inequity. And which is very sensitive to different social classes	
					-simple to calculate	
					-simple to interpret when combine with corresponding curve	
						-the concentration index must be interpreted with the curve
						-does not allow for within or between income group comparisons
Lorenz curve, Gini index	Calculate and compare cumulative percentage of population (ranked by how much resources obtained) and health resource. $G=\sum_{i=1}^{k}P_{i}S_{i+1}-\sum_{i=1}^{k}P_{i+1}S_{i}$	Complex but can aided by statistical software	Systematic assessment, and can be a rough estimation on equity of differences between different regions.	Health resource of individual, total health resource, population of area	-a graphical representation of allocation inequality that can be compared over time and between geographic areas	-incapable of showing different kinds of inequality represented by various shapes of Lorenz curves [34]
					-simple to calculate	
					-data readily available	
					-can be calculated for individual and household level data	
					-easily interpreted when combine with Gini coefficients	
						-does not emphasize inequalities in the top or bottom of the spectrum (polarization)
						-shows the direction of allocation redistribution but does not indicate where the redistributions are occurring
						-does not allow for within or between income group comparisons
						-overlook socioeconomic factors
Theil index	Calculate the equity of health resource by population (area) in each region. $T=\sum_{i=1}^{n}P_{i}\log\frac{P_{i}}{Y_{i}}$	complex	Measure equity of the allocation of health resources between different regions or the units.	Population of units or regions, total population , health resources in units or regions, total resource	-shows the contributions to inequality by within group and between group components	-complex to calculate and interpret.
						-varies greatly when the distribution varies regardless of the change in distribution occurs at the top, middle or bottom
					-high sensitivity to the efficiency of health resource allocation	
						-resource redistributions will impact the calculation irrespective of whether the redistribution takes place between top and bottom or top and middle
						-cannot directly compare populations with different sizes as calculation is dependent on number of individuals in the population or group
Atkinson index	Calculate the health resources of ith region and the proportion of population in which people get the resources. $I_{R}=1-{\left[\sum_{i=1}^{n}{\left(\frac{Y_{i}}{\bar{Y}}\right)}^{1-\varepsilon }f_{i}\right]}^{\frac{1}{1-\varepsilon }}$,if *ε* ≠ 1_,_$I_{R}=1-\exp\left[\sum_{i=1}^{n}f_{i}\log_{e}\frac{Y_{i}}{\bar{Y}}\right]$,if *ε* = 1	complex	Assess the inequity of allocation, address needs of inequity assessment in health benefits analysis	Health resource of ith region, the proportion of population in ith region who get the resource, inequality aversion *ε*	-incorporates a sensitivity parameter directly into the equation.	-sensitivity parameter means that a subjective judgment has been made about inequality
						-not intuitive
Chi-square Value Method	Calculate the actual and theoretical frequency of health resources. $X^{2}=\sum_{i=1}^{k}\frac{{\left(f_{i}-\bar{f_{i}}\right)}^{2}}{\bar{f_{i}}}$	Easy when analyze aided with statistical software	Assess the difference between actual allocation of health resource with the expected allocation	Actual resources in ith region, the total resource, expected frequency of health resource allocation	-sensitive to reflect the inequity of allocation	-always need to standardize the data, otherwise may influence the results
					-reveal the trend of equity over time	
						-the judgment is subjective when based on a certain significance level α
index of dissimilarity	Calculate the health resources and population in each socioeconomic level (region). $\mathrm{ID}=\frac{1}{2}\sum_{j=1}^{k}\left|S_{\mathrm{jh}}-S_{\mathrm{jp}}\right|$	easy	assess the differences of resource allocation in different economic level(region), and calculate the degree of variance	Resource in jth region(or in a certain socioeconomic level), the population in jth region	-can know the differences between the situation of health resource allocation in each region (level) and the proportion of the population in relative region (level)	-can’t reflect the socioeconomic status influence on health resource allocation.
						-not intuitive

Each indicator has its merits and demerits and each serves different purposes. The most commonly used measures are concentration curve combined with concentration index and Lorenz curve combined with Gini index, which are easy to calculate; and intuitive reflection could be made with corresponding curve; concentration index can be used to reflect the unequal distribution caused by socioeconomic factors, however, this measure only calculates income-related inequity without considering the other casual variable and not inequity in health service delivery per se [10]. Gini-coefficient allows direct comparison between units with different size of populations, nevertheless, which overlook socioeconomic status [31]. Calculation of Theil index is complex, however, it can avoid the demerits of uncertainty, imperfection, and incomparability when describe Lorenz curve and calculate Gini index; Theil index can also divide the overall fairness, which can better reflect the differences of distribution within and between groups. Atkinson index has an inequality aversion *ε*, preferences on certain people could be made artificially, and this enables to define how sensitivity the Atkinson index should react to inequalities [35]. Nonetheless Chi-square Value Method is not widely used, it’s convenient to analyze aided by statistical program; which not only can compare the equity condition in different regions, but also can reflect the trend of equity over time. Index of dissimilarity can used to calculate the accurate degree of difference.

### Example: measuring the equity of health resource allocation in Chongqing (China)

We illustrate the measures with health resource data in Chongqing (China) from 1998–2012, here we used Gini-index and Thiel index as examples.

Table 2, for calculating the Gini-index of health resource allocation by population, the Gini-index of beds indicates that allocation of beds was in normal condition from 1998 to 2007, while in 2012 turned to perfect equity condition; allocation of doctor was in perfect equity condition from 1998 to 2012; allocation of nurse was in alert condition from 1998 to 2007, but in 2012, the situation was taking a turn for the better. When calculating the Gini-index of health resource allocation by area, Gini-index were >0.5 from 1998 to 2012, which indicate allocation by area is inequitable for a longtime, and allocation of nurse is especially serious.

**Table 2:** **Gini-index of health resource allocation from 1998-2012**

Year	Allocation by population			Allocation by area		
	Beds	Doctor	Nurse	Beds	Doctor	Nurse
1998	0.3300	0.2373	0.4407	0.5709	0.4934	0.5906
2002	0.3456	0.2728	0.4099	0.5832	0.5273	0.6389
2007	0.3071	0.2725	0.4141	0.5640	0.5326	0.6352
2012	0.2389	0.2843	0.3715	0.5019	0.5494	0.6049

Table 3, the trend of theil-index of health resource allocation from 1998–2012 is nearly as same as Gini-index (Table 2). However, theil-index can decompose the overall equality, which can show the contributions to inequality within group and between group components.

**Table 3:** **Theil-index of health resource allocation from 1998-2012**

Year	Allocation by population			Allocation by area		
	Beds	Doctor	Nurse	Beds	Doctor	Nurse
1998	0.0778	0.0438	0.1149	0.2559	0.1935	0.3195
2002	0.0847	0.0537	0.0980	0.2675	0.2160	0.3362
2007	0.0670	0.0543	0.1108	0.2531	0.2209	0.3315
2012	0.0415	0.0579	0.0859	0.1982	0.2408	0.3013

**Figure 4:**
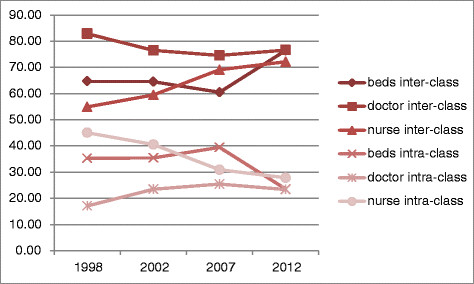
**The contribution rates of difference by population from 1998–2012.**

**Figure 5:**
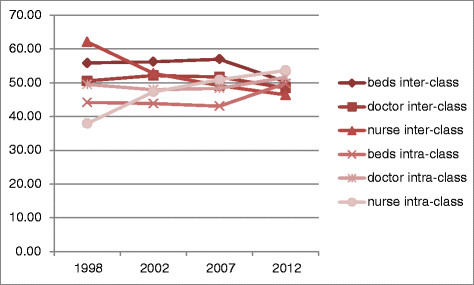
**The contribution rates of difference by area from 1998–2012.**

Figure 4 shows that for health resource allocation by population, the contribution rates of the difference between groups are higher than the difference within group, which indicates that the main reason for inequity in health resource allocation comes from the difference between groups. However, Figure 5, for health resource allocation by area, from 1998–2007, the contribution rates of the difference between groups are a bit higher than the difference within group; while in 2012, the contribution rate of both are almost same, which indicates that inequity comes from difference between groups as well as difference within group (we divided Chongqing into 3 groups, the center of Chongqing, southern Chongqing and northern Chongqing). Theil index can divide the overall fairness, which can better reflect the differences of distribution within and between groups, thus provide targeted advice to policy makers or researchers.

According to the methods of evaluation on horizontal equity of health resource allocation, as well as the availability of the data, we select most commonly used and appropriate methods to study. Sometimes a single index cannot reflect all the allocation disparity problem, you can construct a comprehensive index, or use one of them as key index, and supported by a number of secondary indices, to more comprehensive, in-depth evaluate the equity of health resource allocation.

## Authors’ original submitted files for images

Below are the links to the authors’ original submitted files for images. Authors’ original file for figure 1 Authors’ original file for figure 2 Authors’ original file for figure 3 Authors’ original file for figure 4 Authors’ original file for figure 5
