# Supplementary figures and images for: Methods for measuring horizontal equity in health resource allocation: a comparative study
Source: Health Econ Rev. 2014 Aug 10;4:10. doi: 10.1186/s13561-014-0010-x (PMC4884040; doi:10.1186/s13561-014-0010-x)

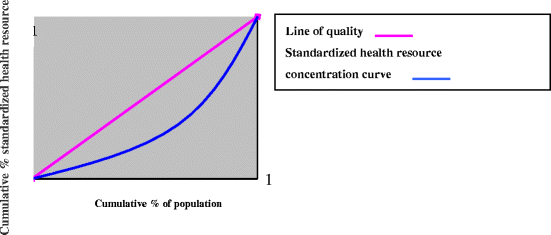

Supplement: Supplementary file 1 — Authors’ original file for figure 1 [file 13561_2014_10_MOESM1_ESM.gif]

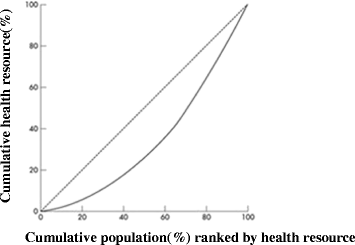

Supplement: Supplementary file 2 — Authors’ original file for figure 2 [file 13561_2014_10_MOESM2_ESM.gif]

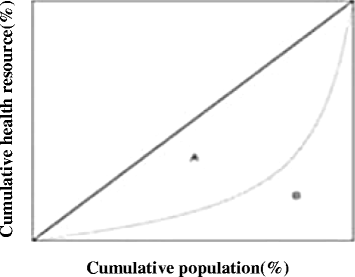

Supplement: Supplementary file 3 — Authors’ original file for figure 3 [file 13561_2014_10_MOESM3_ESM.gif]

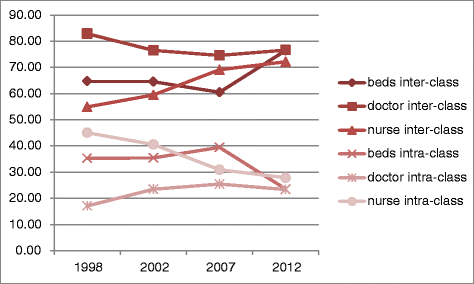

Supplement: Supplementary file 4 — Authors’ original file for figure 4 [file 13561_2014_10_MOESM4_ESM.gif]

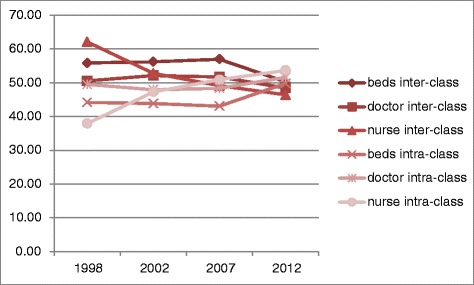

Supplement: Supplementary file 5 — Authors’ original file for figure 5 [file 13561_2014_10_MOESM5_ESM.gif]
